# Discovery of novel inhibitors of human S-adenosylmethionine decarboxylase based on *in silico* high-throughput screening and a non-radioactive enzymatic assay

**DOI:** 10.1038/srep10754

**Published:** 2015-06-01

**Authors:** Chenzeng Liao, Yanlin Wang, Xiao Tan, Lidan Sun, Sen Liu

**Affiliations:** 1 Hubei Key Laboratory of Tumor Microenvironment and Immunotherapy, China Three Gorges University, Yichang 443002, China; 2 College of Medical Science, China Three Gorges University, Yichang 443002, China

## Abstract

Natural polyamines are small polycationic molecules essential for cell growth and development, and elevated level of polyamines is positively correlated with various cancers. As a rate-limiting enzyme of the polyamine biosynthetic pathway, S-adenosylmethionine decarboxylase (AdoMetDC) has been an attractive drug target. In this report, we present the discovery of novel human AdoMetDC (hAdoMetDC) inhibitors by coupling computational and experimental tools. We constructed a reasonable computational structure model of hAdoMetDC that is compatible with general protocols for high-throughput drug screening, and used this model in *in silico* screening of hAdoMetDC inhibitors against a large compound library using a battery of computational tools. We also established and validated a simple, economic, and non-radioactive enzymatic assay, which can be adapted for experimental high-throughput screening of hAdoMetDC inhibitors. Finally, we obtained an hAdoMetDC inhibitor lead with a novel scaffold. This study provides both new tools and a new lead for the developing of novel hAdoMetDC inhibitors.

Natural polyamines (mainly putresine, spermidine, and spermine) are ubiquitous polycationic alkylamines that are required for normal cell growth and development in all eukaryotes and most prokaryotes^1^,^2^,^3^,^4^. A strict regulation of physiological polyamine levels is necessary, and achieved by the combination of synthesis, catabolism, and transport^2^,^4^,^5^,^6^,^7^,^8^,^9^,^10^,^11^,^12^. A rate-limiting reaction in the polyamine biosynthetic pathway is the generation of decarboxylated S-adenosyl-L-methionine (dcAdoMet, or dcSAM) from S–adenosylmethionine (AdoMet, or SAM), which is catalyzed by S-adenosylmethionine decarboxylase (AdoMetDC, or SAMDC; EC 4.1.1.50). AdoMetDC catalyzes the removal of the carboxyl group from AdoMet, and the product dcAdoMet is exclusively used for the biosynthesis of spermidine and spermine^8^,^13^,^14^,^15^,^16^. High levels of polyamines are detected in many human diseases including various tumors, so AdoMetDC has long been an attractive drug target, and a variety of AdoMetDC inhibitors have been developed^8^,^12^,^14^,^15^,^17^,^18^. One AdoMetDC inhibitor, SAM486A (4-amidinoindan-1-one-2**′**-amidinohydrazone, also named as CGP48664), has been shown to be promising in Phase I and II human clinical trials, but the side effects unrelated to the inhibition of AdoMetDC have been observed^19^,^20^,^21^. Therefore, there is great interest to develop more efficacious AdoMetDC inhibitors.

Traditional drug discovery and development, relying on cumbersome experimental synthesis and screening of a large number of compounds, is not only costly but also time consuming. Therefore, the recent years have witnessed the increasing use of time- and cost-saving computer aided drug design (CADD) in lead identification and optimization^22^,^23^,^24^,^25^. One widely adopted strategy in CADD is *in silico* high-throughput (HTP) drug screening based on protein 3D structures, which, to be really fruitful, is generally followed up by complementary experimental HTP screening procedures^26^,^27^,^28^.

To experimentally evaluate the activity of an enzyme, a general method is measuring the change of the products. For example, the activity of ornithine decarboxylase (ODC), which catalyzes another rate-limiting reaction of the polyamine biosynthesis pathway, has been assessed with either non-radioactive or radioactive assays by measuring the product putrescine^29^,^30^,^31^,^32^ or CO_2_^1^,^2^,^3^,^4^. Unlike ODC, however, the evaluation of the activity of AdoMetDC, to our knowledge, has been largely limited to a radioactive assay by measuring ^14^CO_2_ released from S-adenosyl-L-[carboxyl-^14^C]methionine (^14^C-AdoMet)^2^,^4^,^5^,^6^,^7^,^8^,^9^,^10^,^11^,^12^. This radioactive assay is precise, but has a huge limitation due to the involvement of ^14^C-labeled substrates, trapping of ^14^CO_2_, and resource intensive detection procedures. This limitation becomes a burden especially when it comes to experimental HTP screening of AdoMetDC inhibitors^8^,^13^,^14^,^15^,^16^. Although the high-performance liquid chromatography (HPLC) analysis of the other product, dcAdoMet, is an effective alternative method^8^,^12^,^14^,^15^,^17^,^18^,^33^, it is also quite complicated and not suitable for HTP screening. Thus,, the lack of an easy-to-use enzymatic assays has largely hampered the development of novel AdoMetDC inhibitors.

In this paper, we report the screening of a novel hAdoMetDC inhibitor lead by integrated computational and experimental HTP assays. Firstly, we describe a simple, inexpensive, nonradioactive, and quantitatively acceptable spectrophotometric assay for assessing the enzymatic activity of hAdoMetDC *in vitro*. Secondly, we present a computational HTP protocol for screening hAdoMetDC inhibitors based upon a structure model of hAdoMetDC. Finally, we show that the presented spectrophotometric assay, as a complementary assay to the computational protocol, could be adapted for the HTP screening of hAdoMetDC inhibitors. Altogether, this paper describes a novel HTP pipeline for screening hAdoMetDC inhibitors *in silico* and *in vitro*, as well as the discovery of an hAdoMetDC inhibitor lead with a novel scaffold.

## Results

### The spectrophotometric assay for assessing hAdoMetDC activity

**Figure 1:**
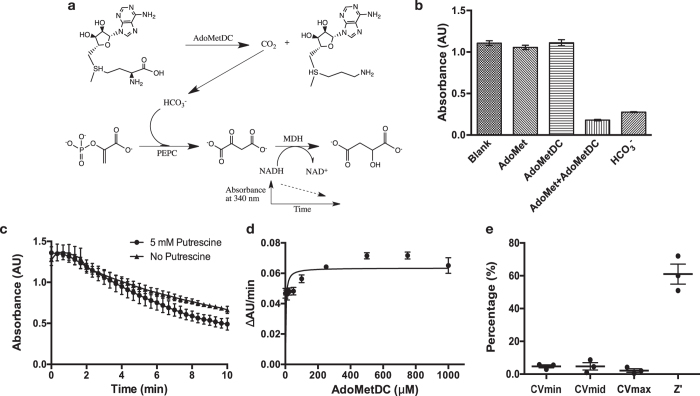
The AdoMetDC-PEPC-MDH assay is qualitatively and quantitatively applicable. (**a**) The scheme of the reaction mechanism of this assay. (**b**) The PEPC-MDH assay detected the activity of hAdoMetDC, which was only seen when the substrate (AdoMet) and the enzyme co-existed. HCO_3_^-^ is the positive control buffer (NaHCO_3_). (**c**) With 5 mM of putrescine in the reaction buffer, the activity of hAdoMetDC was slightly higher than without putrescine. (**d**) The kinetic parameters were determined by the assay. The concentrations of the substrate AdoMet were 0, 5, 10, 25, 50, 100, 250, 500, 750, and 1000 μM. The data are shown in means with standard deviations (3 replications), and fitted with the Michaelis-Menten equation. (**e**) The signal variability data for assessing hAdoMetDC activity were calculated according to the HTS Assay Validation protocol in the reference^40^. CVmin, CVmid, and CVmax were the calculated cross validation values for 0% activity, 50% activity, and 80% activity, respectively. The data were calculated from three independent experiments, and are shown as dots. The mean and S.E.M. values are shown as lines.

AdoMetDC catalyzes the removal of the carboxyl group from AdoMet to produce dcAdoMet and CO_2_. In weak basic solutions, low-level CO_2_ exists primarily as bicarbonate (HCO_3_^-^). The PEPC-MDH method was widely used for detecting CO_2_ produced by decarboxylases (ODC, for example)^1^,^19^,^20^,^21^,^32^,^34^,^35^,^36^,^37^,^38^,^39^. In this method, the first step, catalyzed by Phosphoenolpyruvate Decarboxylase (PEPC), is the bicarbonate condenses with phosphoenol pyruvate to form oxalate, and in the second step, oxalate is enzymatically reduced by Malate Dehydrogenase (MDH, using an NADH cofactor) to form malate and NAD^+^. At 340 nm, NADH absorbs light but NAD^+^ does not, so the decrease in light absorbance can be used to evaluate the presence of CO_2_ in the reaction system (Fig. 1a).

Since the PEPC-MDH method has never been used in measuring hAdoMetDC activity, we first tested whether this method could be applicable or not. As shown in Fig. 1b, the hAdoMetDC activity could be detected using this method. Nevertheless, we noticed that AdoMet caused the decrease of the absorbance value. We then checked the background effect of AdoMet, and found that the optimal concentration of AdoMet should be not higher than 1.0 mM (Supplementary Fig. S1). Therefore, the concentration of AdoMet used in our later experiments was 1.0 mM, unless indicated otherwise, since lower AdoMet concentration produces fewer CO_2_. The concentration of hAdoMetDC was 1.0 μM, which was not varied since it showed stable results. So the AdoMetDC-PEPC-MDH assay (Fig. 1a) could be applicable.

Putrescine was reported to be able to stimulate the activity of hAdoMetDC^5^,^22^,^23^,^24^,^25^,^41^, so we tested the role of putrescine with this assay. Firstly, according to the reported data, we tested different concentrations of putrescine to evaluate the possible background effect. Our data showed that at the tested concentrations (0.0–5.0 mM), putrescine had no obvious interference on the light absorption (Supplementary Fig. S2). As shown in Fig. 1c, this assay was able to detect the stimulation potential of putrescine on hAdoMetDC, but the difference was quite small. A reason could be that this assay was not sensitive enough, since putrescine stimulates hAdoMetDC activity only up to 2 folds^5^,^26^,^27^,^28^,^41^. Therefore, for simplicity, we did not add putrescine in our later experiments.

Then we tried to determine the kinetic parameters (*K*_m_ and *k*_cat_) of hAdoMetDC with this assay. Based on the results from the radioactive assay in previous reports, when without putrescine, the *K*_m_ value of hAdoMetDC varied from 60 μM to 320 μM^29^,^30^,^31^,^32^,^42^,^43^, and *k*_cat_/*K*_m_ was 2.5 × 10^4^ M^−1^/s^41^. By varying the concentration of AdoMet, we were able to get similar results, with *K*_m_ being 3.1 ± 1.8 μM, and *k*_cat_/*K*_m_ being 2.0 × 10^4^ M^−1^/s (Fig. 1d,e).

In conclusion, the AdoMetDC-PEPC-MDH assay is applicable and comparable to the radioactive assay, although the sensitivity might be slightly lower.

### Computational HTP screening of hAdoMetDC inhibitor

AdoMetDC is expressed as a single chain proenzyme in cells at first, and then auto-processed to form the active enzyme^10^,^44^,^45^. The active form of hAdoMetDC has two chains, the beta chain (residues 1-67), and the alpha chain (residues 68-334), with residue Ser68 being converted to a pyruvoyl group.

**Figure 2:**
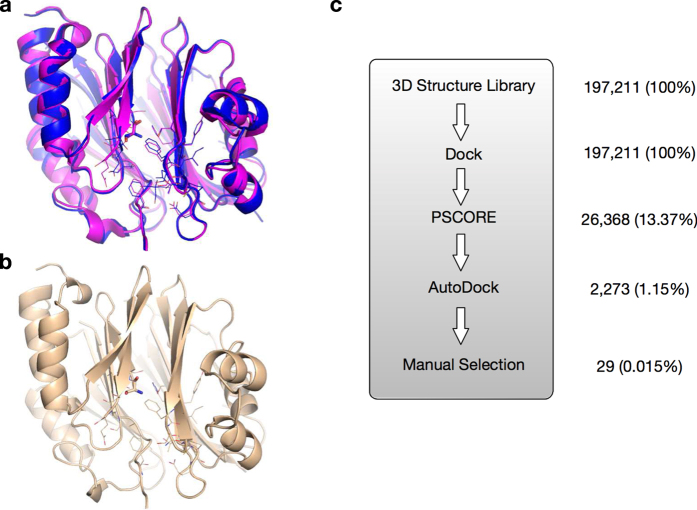
(**a**) The structural comparison of an inhibitor binding state of hAdoMetDC with the residue 68 being the pyruvoyl group (PDB ID: 3DZ5, colored in magentas), and a mutant state with Ser68 intact (PDB ID: 1JL0, colored in blue). The important residues forming the substrate/inhibitor binding pocket are shown in lines, and the residue 68 in sticks. (**b**) The modified and optimized structure of the model (colored in gold) used in the computational HTP screening. This model was constructed by substituting the pyruvoyl group 68 in 3DZ5 with Ser68 in 1JL0. (**c**) The brief computational HTP screening scheme. The filtering efficacies are shown in molecule numbers and percentages (in parentheses).

Comparing the available X-ray structures of hAdoMetDC, we noticed that the conformation of the pyruvoyl group was very similar to the un-converted serine (Fig. 2a). Therefore, considering the compatibility of the general computational tools on non-standard residues, we decided to use a modified structure of hAdoMetDC for *in silico* inhibitor screening. In this structure, the pyruvoyl group in 3DZ5 (PDB ID) was substituted with the Ser68 in 1JL0 (PDB ID), a mutant hAdoMetDC preventing the conversion of Ser68 to the pyruvoyl group (Fig. 2b).

The virtual screening process was similar to Wu *et al.*^46^ with some modifications (Fig. 2c). Briefly, 26,368 of the 197,211 molecules passed the Pscore^47^ filter, among which 2,273 passed the following Autodock^48^ filter. The Autodock filtering rules are: (1) the lowest predicted score is lower than -7.5 (the Score Rule); (2) 75% of the output conformations are within 3.0 Å (RMSD) from the lowest-score conformation (the Cluster Rule); (3) the average score contribution of each heavy atom is better (lower) than -0.28 (the Score Density Rule). Finally, these 2,273 molecules were manually checked, and 29 molecules were selected for experimental screening (26 of them were available for purchase from SPECS, http://www.specs.net; Supplementary Table S1), according to the following three rules: (1) less than 30% of the molecule structure, especially hydrophobic groups, is out of the pocket; (2) at least one of the top 5 conformations fits the pocket well; (3) only the molecule with lower score was chosen if two molecules have similar conformations.

### Experimental screening of hAdoMetDC inhibitor

**Figure 3:**
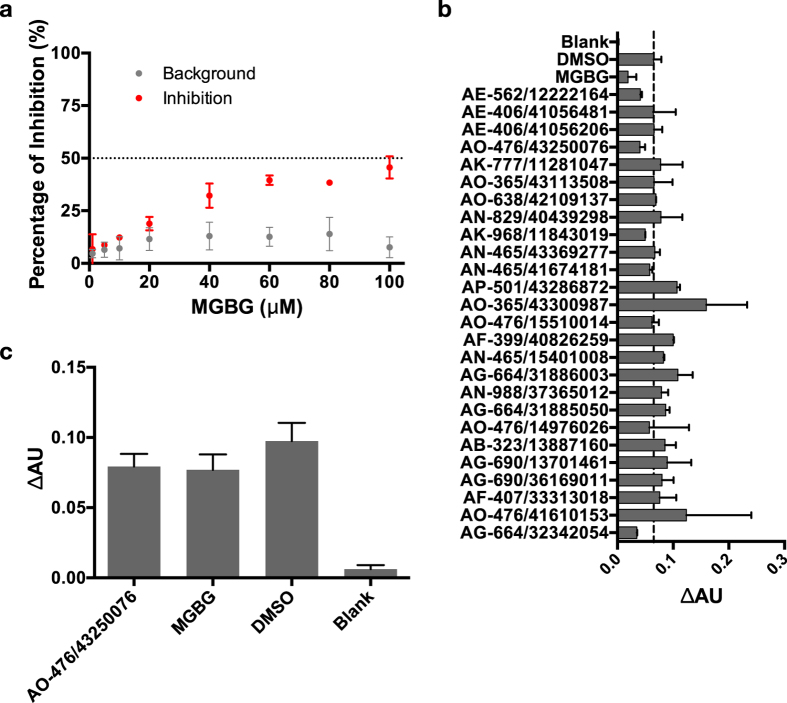
(**a**) The background effects and the inhibition potencies of different concentrations of MGBG were determined with the AdoMetDC-PEPC-MDH assay. The inhibition percentage of 100 μM of MGBG is around 50%. (**b**) The AdoMetDC-PEPC-MDH assay was used to screening hAdoMetDC inhibitors based on the computational HTP screening results. MGBG was added as a positive control. The concentrations of the drugs were 100 μM. The compounds are named by SPECS IDs. (**c**) AO-476/43250076 was confirmed to have inhibitory potency comparable with MGBG. The data are shown in means with standard deviations (3 replications). DMSO was the positive control without compounds, and the blank control was the sample without hAdoMetDC.

To test the possible use of the AdoMetDC-PEPC-MDH assay in hAdoMetDC inhibitor screening, we firstly used a known inhibitor of AdoMetDC, methylglyoxal bis(guanylhydrazone) (MGBG), as a positive control. The background effect of MGBG was checked (Fig. 3a, Supplementary Fig. S3a), and the upper-limit concentration was determined to be 100 μM to maximally avoid signal interference. Although this has resulted in the IC_50_ value of MGBG not being able to be quantitatively determined in this assay, the 50% inhibition concentration was shown to be around 100 μM (Fig. 3a). This value was comparable to the reported IC_50_ value of MGBG (45 μM) in the absence of putrescine determined by the radio-activity assay^49^, considering the sensitivity difference between the assays.

To showcase the possible application of the AdoMetDC-PEPC-MDH assay in the experimental HTP screening of novel AdoMetDC inhibitors, the 26 compounds from the computational HTP screening, along with MGBG as a positive control, were experimentally screened. In the first round of screening, several compounds showed inhibition signals (Fig. 3b). After checking the background absorbance of the compounds and excluding the possible inhibition of the PEPC-MDH steps, we noticed that one compound, AO-476/43250076 (SPECS ID), showed comparable inhibitory effects as MGBG (Fig. 3c, Supplementary Fig. S3b). In addition, MGBG showed stable inhibition results throughout the screening process.

### Structural analysis of the identified inhibitor

**Figure 4:**
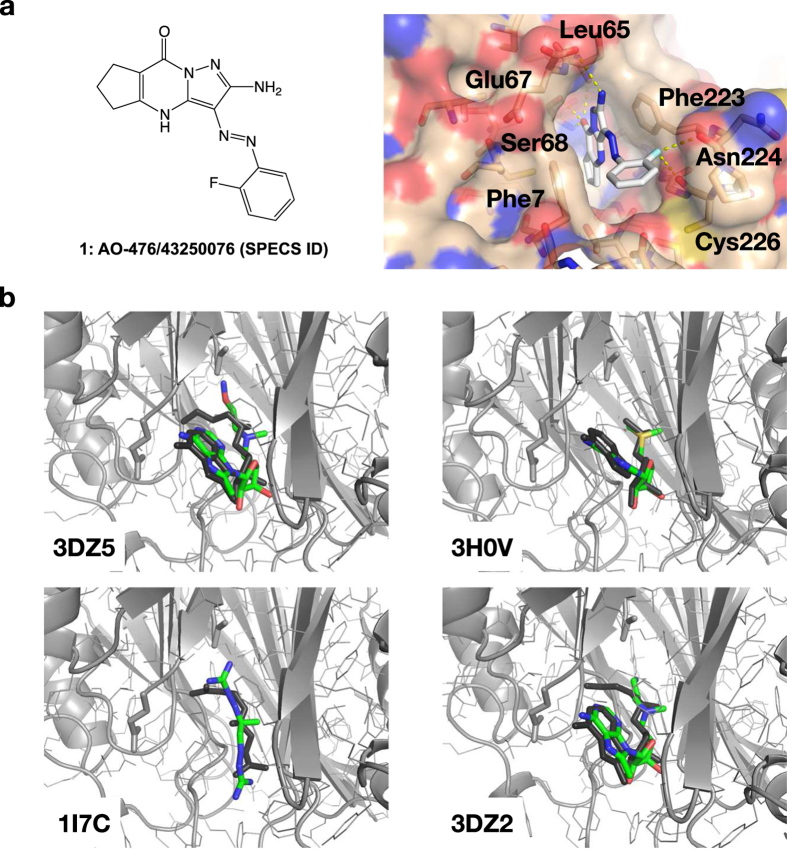
(**a**) The 2D structure (left) and the docked conformation (right) of AO-476/43250076 in the pocket of hAdoMetDC. In this study, Ser68 of hAdoMetDC was kept unchanged for better compatibility with the computational protocols. The residues forming potential polar interactions with the small molecule are labeled. (**b**) The comparison of the docked conformations of known inhibitors and the X-ray conformations. These known inhibitors were computationally docked to the modified hAdoMetDC. The un-modified active form of hAdoMetDC (PDB ID: 3DZ5) is shown here in grey cartoon and used for structure alignment only, and the residues 67 and 68 are shown in sticks. The computationally docked models of the known inhibitors are shown in black, and the X-ray conformations are colored by atoms. Only four representative models are shown here, and more models are shown in Supplementary Fig. S4. The PDB IDs are marked on the figures for corresponding inhibitors.

As shown in Fig. 4a, in the docked model, AO-476/43250076 forms quite nice interactions with hAdoMetDC. The major interactions are the ring-ring stacking interactions with Phe7 and Phe223, the polar interactions with the side-chain or main-chain atoms of Leu65, Ser68, Glu67, Asn224, and Cys226. These interactions are similar to those found in known inhibitors^15^, except Ser68, which was modified in this study.

In the docked model, Ser68 has several hydrogen bond interactions with the small molecule. Although the conformation of this residue is similar to the pyruvoyl group in the active AdoMetDC (Fig. 2a), the serine provides more side-chain hydrogen bonds due to the additional amine group. Since we only got a quite weak inhibitor in this small-scale screening, we were wondering if this difference dramatically distorted the screening result. Therefore, we docked known hAdoMetDC inhibitors with complex structures available to the modified hAdoMetDC model in this study. As shown in Fig. 4b and Supplementary Fig. S4, the docking protocol was able to nicely recapture the binding conformations of those known inhibitors in the X-ray structures, although the X-ray structures were acquired using the active form of AdoMetDC with the Ser68 being the pyruvoyl group. Nonetheless, we noticed that, when an inhibitor had direct interactions (Schiff base) with the pyruvoyl group in the X-ray structure (PDB ID: 3DZ5, 1I7B, 1I7M), the docked conformation was quite different within the involved groups, even the other parts of the molecule were very close to the crystal structure. But when the inhibitor did not directly interact with the pyruvoly group in the crystal structure (PDB ID: 3H0V, 3H0W, 3DZ4, 3DZ6), the docked conformation was very close to the crystal structure. The docking result of the low-affinity linear-chain inhibitor MGBG (PDB ID: 1I7C) was not as good as the others, suggesting that forming ring-ring stacking interactions with Phe7 and Phe223 was important for high affinity. Another exception is the inhibitor in 3DZ2 (PDB ID), for which one terminal part was very different between the docked model and the crystal structure, although that part did not interact directly with the pyruvoyl group in the crystal structure. However, we noticed that this inhibitor had the lowest affinity in these inhibitors, and the crystal structure was quite different from the other similar structures too.

Based on these results, we propose that the modified structure in this study (by substituting the pyruvoyl group with serine) was good for computational screening of AdoMetDC inhibitors, and it should be good to experimentally screen more compounds from the computational hits. Considering most current computational tools only parameterize standard residues, we expect this modified model could serve as a better model than the active form structure with the pyruvoyl group.

## Discussion

AdoMetDC is a rate-limiting enzyme in the polyamine biosynthetic pathway. It catalyzes the conversion of AdoMet to dcAdoMet (Fig. 1a), and the latter is exclusively utilized for providing propyl amines for the synthesis of spermidine and spermine^8^,^14^,^15^,^16^. Therefore, AdoMetDC can be inhibited to decrease the level of polyamines in cancerous cells, and one AdoMetDC inhibitor, SAM486A, showed promising results in clinic trials^19^,^20^,^21^. However, side effects and disappointing results in some studies from the known AdoMetDC inhibitors have prompted researchers to develop novel ones^13^,^50^.

AdoMetDC catalyzes the decarboxylation of AdoMet to produce CO_2_, which was previously measured by a radioactive assay^5^,^7^,^9^,^10^,^11^. A widely used method for detecting CO_2_, the PEPC-MDH method, was suitable for decarboxylases including ODC, another important enzyme in the polyamine pathway. But surprisingly, this method has never been reported to be applicable in measuring the activity of AdoMetDC. Recently, Smithson *et al.*^1^ presented an optimized protocol based on this method for screening inhibitors of decarboxylases in the polyamine pathway, and they still used ODC as an example, although the authors claimed that it should be suitable for AdoMetDC too. This is quite surprising to us, since the PEPC-MDH method has actually been used on ODC in many previous studies^32^,^37^,^38^,^39^. Therefore, we supposed that some conditions could have hampered the use of this method in assessing AdoMetDC activity. Nevertheless, this method, compared to the radiometric assay and the occasionally used HPLC assay, has more advantages, such as simpler, faster, non-radioactive, inexpensive, and virtually suitable for any biochemistry laboratories. So we set out to see if this method could be optimized to evaluate the activity of AdoMetDC, either qualitatively or quantitatively.

**Figure 5:**
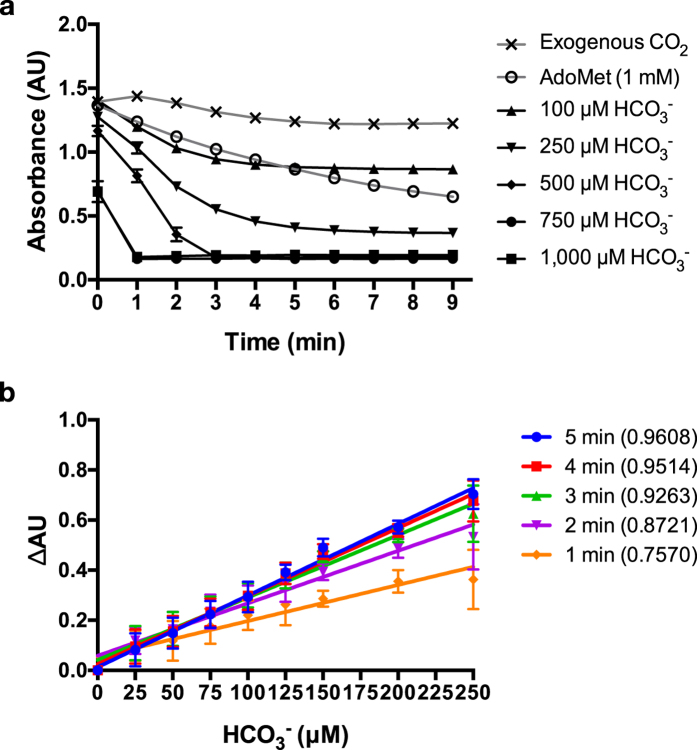
(**a**) The time-dependent effects of exogenous CO_2_ and different concentrations of HCO^3-^ (positive control). In the first 5 minutes, the exogenous CO_2_ had limited interference in this assay, and 1 mM of AdoMet (the highest concentration used in this study) caused absorbance changes close to 100 μM of HCO_3_^-^. (**b**) The linear ranges of the detection concentration of the product (HCO_3_^-^ as reference) and the sampling time spans were examined. When the total concentration of CO_2_ (the reaction product and the exogenous) is lower than 250 μM, the absorbance changes (by subtracting the absorption values of 0 min and the indicated time points) quantitatively reflected the concentration change. The R square values of the linear fitting are shown in the parentheses. The data are shown in means with standard deviations (3 replications).

To minimize the interference of exogenous CO_2_ from air and buffer solutions, a possible way is to perform this assay under nitrogen atmosphere, like what Smithson *et al.*^1^ did in their study. This could be better, but it increases the complexity of the assay, and is not applicable to less-equipped biochemistry laboratories. Meanwhile, the perturbation of exogenous CO_2_ should be very small, since the other studies did not use nitrogen atmosphere in similar tests^32^,^34^,^35^,^37^,^38^,^39^. Therefore, we evaluated the interference of exogenous CO_2_. As shown in Fig. 5a, during the measuring time span (0–5 min), the exogenous CO_2_ did cause the decrease of the light absorbance. But, the decrease was quite small and slow. Actually, although the nitrogen atmosphere made this decrease slower, it could not totally eliminate it^1^. Furthermore, the decrease was linear and within the linear range of the assay (Fig. 5b), and therefore could be justified by subtracting a background control. So we conclude that, at least in a short time range (5 min), it is acceptable to do this assay in open laboratory environment, which is important since it will not compromise the easy implementation of this assay.

To apply this method to the assessment of AdoMetDC activity, we first checked the possible false-positive/negative effects from different components (Supplementary Fig. S1, S2) in the AdoMetDC reaction. We noticed that AdoMet caused remarkable decrease of the absorbance at 2.0 mM or higher concentrations. But the other commonly used component, putrescine, did not significantly affect the absorbance under working concentrations (Supplementary Fig. S2). To be simple, we used 1.0 μM of AdoMetDC in all experiments, and it did not show adverse effects too (Fig. 1b). Therefore, we limited AdoMet to 1.0 mM or less in our following experiments. According to the reaction stoichiometry (Fig. 1a), it could produce the same concentration of CO_2_ at the most, which is in the linear range of the assay (Fig. 5a,b).

We then used this assay to evaluate the activity of hAdoMetDC. As shown in Fig. 1b, this assay qualitatively detected hAdoMetDC activity sensitively. Moreover, this assay quantitatively determined hAdoMetDC activity parameters (*K*_m_ and *k*_cat_/*K*_m_) (Fig. 1d), which are comparable to the data from the radiometric assay^41^,^42^,^43^. So we conclude that the AdoMetDC-PEPC-MDH assay proposed in this study is suitable for evaluating AdoMetDC activity and the sensitivity of this assay in evaluating AdoMetDC activity is reasonable, since a gentle activity stimulation (~2 × by *k*_cat_/*K*_m_)^5^,^41^ of AdoMetDC by putrescine was distinguishable (Fig. 1c).

Having this simple assay available, we decided to test its potential in inhibitor screening by coupling to *in silico* HTP screening. Previously, Brooks *et al.*^13^ reported a virtual screening study of hAdoMetDC. They found NSC 354961 could inhibit hAdoMetDC with a low micromolar IC_50_ value; however, we noticed that the same compound is able to inhibit telomerase^51^ and anthrax lethal factor^52^ with similar potential, which raises the concern about the specificity of this compound. Moreover, Brooks *et al.* computationally screened a small library containing only 1,990 compounds. Therefore, we hoped to find novel hAdoMetDC inhibitor leads by screening a larger compound library, and chose a SPECS compound library containing 197,211 molecules (November 2009 version for 10 mg, http://www.specs.net) prepared by Wu *et al.*^46^.

As for the protein structure, we noticed three different forms of hAdoMetDC in the PDB database ( http://www.pdb.org): the active form with the residue 68 converted to the pyruvoyl group (PDB ID 3DZ5 for example), the intact form before the auto-cleavage (PDB ID 1MSV, the S48A mutant), and an alternative form (PDB ID 1JL0, the H243A mutant; the Glu67-Ser68 peptide bond is cleaved but Ser68 is not converted to the pyruvoyl group). The pyruvoyl group is necessary for the activity of AdoMetDC^44^,^53^, but as a non-standard residue, it is not compatible with most current computational tools, which hampers the implementation of computer aided drug design (CADD) on this key enzyme. By analyzing the available X-ray structures, we noticed that Ser68 in the intermediate form (PDB ID 1JL0, the H243A mutant) has a conformation similar to that of the pyruvoyl group in the active form (PDB ID 3DZ5, wild-type) (Fig. 2a). Therefore, we supposed that this similarity could be taken advantage of to improve the compatibility of the hAdoMetDC structure in computational tools. Thus, we substituted the pyruvoyl group in the active form structure (PDB ID 3DZ5) with the Ser68 in the intermediate form structure (PDB ID 1JL0) to get a modified structure model (Fig. 2b), which was then used in the *in silico* screening in this study. To investigate how this modification might distort the inhibitor binding potential, known hAdoMetDC inhibitors were computationally docked to this modified structure. Interestingly, the docked conformations of those known inhibitors were recaptured very well (Fig. 4b, Supplementary Fig. S4). This analysis suggests that this modified structure is suitable to be used in the computational screening of hAdoMetDC inhibitors. Nonetheless, as indicated by the discrepancies between the docked and the X-ray conformations in some cases, the difference between serine and the pyruvoyl group should be further investigated in later optimizations. For example, the pyruvoyl group can form covalent bonds with covalent inhibitors, so the substitution by serine in this study might miss those potential covalent leads.

Using a similar screening pipeline as Wu *et al.*^46^ with some modifications (Fig. 2c), we screened out, selected and purchased 26 compounds for experimental validation using the presented AdoMetDC-PEPC-MDH assay. As shown in Fig. 3a–c, in addition to the control inhibitor MGBG, one compound (AO-476/43250076) was found to be able to inhibit hAdoMetDC. The docked model showed that AO-476/43250076 binds the enzyme with interactions similar to known inhibitors^15^. The scaffold of this compound is quite different from previously known AdoMetDC inhibitors^13^,^15^,^54^, and therefore could represent a novel class of AdoMetDC inhibitors. The relatively low potency of this compound indicates that further optimization is necessary, and enlightened by the docking analysis of the known inhibitor in this study, a reasonable way is to add a functional group that could form a Schiff base to the active site pyruvoyl group.

From this showcase of the potential of the AdoMetDC-PEPC-MDH assay, we believe this assay can be readily used in more intensive experimental HTP screening. We also want to point out that, although background interference could come from the absorbance of the compounds or the unexpected inhibition of the PEPC-MDH system, this experimental assay still has incomparable advantages (simple, inexpensive, nonradioactive, and widely applicable) against the complicated radioactive or HPLC assays used before. Meanwhile, as shown in this study, these backgrounds could be fairly justified or even eliminated by additional control experiments, or by adjusting the concentration of the tested compound. As a possible improvement or complement, the NADH fluorescence could be used (Supplementary Fig. S5) to confirm that the compound is indeed effective and the PEPC-MDH system is not inhibited by chance. Therefore, we feel that this assay would accelerate the discovery of novel AdoMetDC inhibitors, considering most known AdoMetDC inhibitors are based on the deoxyadenosine group^15^,^54^.

Taken together, we developed a simple, non-radioactive, time- and cost-saving assay for evaluating the activity of hAdoMetDC. We showed that this assay was both qualitatively and quantitatively acceptable in assessing hAdoMetDC activity, or in evaluating and screening hAdoMetDC inhibitors. We also showed that, by substituting a non-standard residue, the pyruvoyl group, with a standard residue (serine), the hAdoMetDC structure could be used as a fair target for inhibitor screening. This strategy might be also applicable for the other pyruvoyl-dependent enzymes. Finally, an inhibitor lead of hAdoMetDC with a novel scaffold was identified and validated with the computational and experimental protocols presented in this study. Due to the homologous feature of different AdoMetDCs, we suppose that these assays should be readily used on other AdoMetDCs (AdoMetDCs in parasites, for example). At last, we hope the AdoMetDC-PEPC-MDH assay established in this paper would free researchers from cumbersome radioactive or HPLC assays, and helps accelerating the study and drug development of AdoMetDC.

## Methods

### *In silico* high-throughput screening

The modified hAdoMetDC structure used in this study was prepared by substituting the pyruvoyl group (residue ID: 68) of an active structure (PDB ID: 3DZ5) with Ser68 in an in-active structure (PDB ID: 1JL0) after the protein backbones were aligned in Pymol^55^. Then the modified structure was optimized in Rosetta (version: 3.5)^56^ by side-chain repacking and energy minimization. The Rosetta-optimized structure was then used to define the binding pocket with LigBuilder (version: 2.0)^57^ and Pocket (version: 3.1)^58^. After that, the structure was submitted for docking in Dock (version 4.0)^59^ against a SPECS small molecule library containing 197,211 structures (November 2009 version for 10 mg, http://www.specs.net); 3D structures prepared by Wu *et al.*^46^), and the docked results were evaluated with PScore^47^. The filtered small molecules and the modified structure of hAdoMetDC were docked again using AutoDock Vina (version: 1.1.2)^48^ before expert checking.

### Docking analysis of known inhibitors

The complex structures of hAdoMetDC and inhibitors were downloaded from the PDB database ( http://www.pdb.org), and the structures of the inhibitors were separated. Then the inhibitors were docked to the modified hAdoMetDC structure in AutoDock Vina as above. To eliminate the X-ray structural information, all rotatable bonds of the inhibitors were allowed to rotate freely. Finally, the top 5 conformations by AutoDock score were evaluated and compared to the original X-ray conformation.

### Protein expression and purification

The full-length coding sequence of hAdoMetDC proenzyme (NCBI Reference Sequence: NP_001625.2) was inserted in pET-15b (Novagen) to make the pET-15b/hAdoMetDC plasmid using the BamH I/Nde I digestion sites^60^. This plasmid was verified by DNA sequencing, and then transformed into the Escherichia coli strain BL21(DE3) for induced expression as a 6× His tagged product with 0.5 mM of IPTG (isopropyl β-D-1-thiogalactopyranoside) for 12 hours at 15 ˚C, 250 rpm. The cells were collected by centrifugation, resuspended in the lysis buffer (20 mM Na_2_HPO_4_, 500 mM NaCl, 2.5 mM putrescine, 0.02% Brij-35, 10 mM imidazole, pH 7.0), and broken by sonication. The cell lysate was clarified by centrifugation, and the supernatant was loaded to a HisTrap HP (GE Healthcare) column for affinity capture of the His-tag hAdoMetDC. The His-tag hAdoMetDC was eluted with 150 mM imidazole in the lysis buffer, and then subjected to a further purification with a Superdex 75 size-exclusion column (GE Healthcare) in the storage buffer (20 mM Na_2_HPO_4_, pH 7.0, 500 mM NaCl). The final product was collected and analyzed with 15% SDS-PAGE. The 6× His tag was not cleaved off in this study.

### Enzymatic activity assay

The carbon dioxide kit was purchased from BioSino Bio-technology and Science Inc (Beijing, China). This kit contains the reagent R1 [7.0 mM pohosphoenolpyruvate (PEP), 8.0 mM MgCl_2_], R2 [400 unit/L PEP carboxylase (PEPC), 600 unit/L malate dehydrogenase (MDH), 0.45 mM NADH], and the calibration standard (25 mM NaHCO_3_). Before detection, R1 and AdoMet were mixed in one cell of a 96-well plate, R2 and hAdoMetDC in the other cell. Then the reaction was initiated by transferring R1/AdoMet to R2/hAdoMetDC and the absorbance data were recorded at 340 nm, 37 °C for up to 10 minutes on Multiskan Spectrum (Thermo Scientific). The final reaction mixture was 200 μL, including 143 μL R1, 47 μL R2, 1 mM AdoMet, and 1 μM hAdoMetDC. The concentrations of AdoMet and hAdoMetDC were adjustable according to different experiments. The absorbance data of the starting time and 5 min were subtracted unless noted otherwise.

### Inhibition assay

To measure the effect of AdoMetDC inhibitors, the indicated inhibitor was mixed with R1/AdoMet before detection. For inhibitors dissolved in DMSO, DMSO was also added in corresponding blank controls. The other steps were similar to the enzymatic activity assay. The inhibition percentage was calculated as [1 - ΔAU(with inhibitor)/ΔAU(without inhibitor))] × 100%.

### Experimental high-throughput screening assay

To screen multiple compounds in parallel, all compounds were firstly adjusted to same concentrations, and a final concentration of 100 μM was used in the initial screening for all compounds. The following steps were similar with the inhibition assay.

### Background checking assay

To check the background effects of inhibitors (compounds), different concentrations of inhibitors (compounds) were added to the reaction system similar as the enzymatic activity assay, except that hAdoMetDC was not included, or the NaHCO_3_ standard was used. This assay was applied to check the background absorption of the small inhibitors (compounds), and the possible inhibition of the PEPC-MDH assay.

## Additional Information

**How to cite this article**: Liao, C. *et al.* Discovery of novel inhibitors of human S-adenosylmethionine decarboxylase based on *in silico* high-throughput screening and a non-radioactive enzymatic assay. *Sci. Rep.* **5**, 10754; doi: 10.1038/srep10754 (2015).

## Supplementary Material

Supplementary Information
