# Supplementary material for: Discovery of novel inhibitors of human S-adenosylmethionine decarboxylase based on in silico high-throughput screening and a non-radioactive enzymatic assay
Source: Sci Rep. 2015 Jun 1;5:10754. doi: 10.1038/srep10754 (PMC5377238; doi:10.1038/srep10754)
Supplement: Supplementary Information [file srep10754-s1.pdf]

Discovery of novel inhibitors of human S-adenosylmethionine decarboxylase based on *in silico* high-throughput screening and a non-radioactive enzymatic assay

Chenzeng Liao[1,2], Yanlin Wang[1,2], Xiao Tan[1,2], Lidan Sun[1,2], Sen Liu[1,2,*]

[1]Hubei Key Laboratory of Tumor Microenvironment and Immunotherapy, China Three Gorges University, Yichang 443002, China
[2]College of Medical Science, China Three Gorges University, Yichang 443002, China

Supplementary Table S1. Experimentally screened compounds in this study.

SPECS ID-number	Chemical Name
AG-664/32342054	2-(6H-indolo[2,3-b]quinoxalin-6-yl)acetohydrazide
AO-476/41610153	7,10-dioxo-4,5-dihydro-7H,10H-pyrano[3,2-c]pyrrolo[3,2,1-ij]quinolin-8-yl acetate
AF-407/33313018	2,3,4-pentanetrione 3-[(2-methyl-1H-benzimidazol-6-yl)hydrazone]
AG-690/36169011	N-{4,6-bisnitro-2-oxo-2,3-dihydro-1H-benzimidazol-5-yl}acetamide
AG-690/13701461	2-[(7-butyl-3-methyl-2,6-dioxo-2,3,6,7-tetrahydro-1H-purin-8-yl)sulfanyl]acetamide
AB-323/13887160	6-nitro-1-isopropylpyrido[2,3-d]pyrimidine-2,4(1H,3H)-dione
AO-476/14976026	1-ethoxy-3-methylpyrido[1,2-a]benzimidazole-4-carbonitrile
AG-664/31885050	N'-(5-hydroxy-1,3-dimethyl-2,4-dioxo-1,2,3,4-tetrahydropyrido[2,3-d]pyrimidin-7-yl)acetohydrazide
AN-988/37365012	2-[(1,3,7-trimethyl-2,6-dioxo-2,3,6,7-tetrahydro-1H-purin-8-yl)sulfanyl]acetamide
AG-664/31886003	2-cyano-N'-(5-hydroxy-1,3-dimethyl-2,4-dioxo-1,2,3,4-tetrahydropyrido[2,3-d]pyrimidin-7-yl)acetohydrazide
AN-465/15401008	3-(1,3-dimethyl-2,6-dioxo-1,2,3,6-tetrahydro-7H-purin-7-yl)propanenitrile
AF-399/40826259	ethyl 3-(2-hydroxyethyl)-6-methyl-3,4-dihydro-2H-furo[3',2':3,4]naphtho[2,1-e][1,3]oxazine-5-carboxylate
AO-476/15510014	3-methyl-1-(1H-1,2,4-triazol-5-ylsulfanyl)pyrido[1,2-a]benzimidazole-4-carbonitrile
AO-365/43300987	N,N-dimethyl-N-{4-[6-(3-pyridinyl)[1,2,4]triazolo[3,4-b][1,3,4]thiadiazol-3-yl]phenyl}amine
AP-501/43286872	3-(2-fluorobenzyl)-6-(3-pyridinyl)[1,2,4]triazolo[3,4-b][1,3,4]thiadiazole
AN-465/41674181	[1,2,4]triazolo[3,4-b][1,3]benzothiazole-3-sulfonic acid
AN-465/43369277	4-(2-{[(5-phenyl-2-furyl)methyl]amino}ethyl)benzenesulfonamide
AK-968/11843019	2-[({3-nitro-1H-1,2,4-triazol-1-yl}acetyl)amino]-4,5-dimethyl-3-thiophenecarboxamide
AN-829/40439298	6,8-dinitro-2,4-dihydro-1H-[1,2,4]triazolo[3,4-c][1,4]benzoxazin-1-one
AO-638/42109137	6H-pyrrolo[3,4-f][1,10]phenanthroline-5-carboxylic acid
AO-365/43113508	6-[(4-methylphenoxy)methyl]-3-(3,4,5-trimethoxyphenyl)[1,2,4]triazolo[3,4-b][1,3,4]thiadiazole
AK-777/11281047	4-amino-5-methoxy-6-phenyl-2,3-pyridinedicarbonitrile
AO-476/43250076	2-amino-3-[(2-fluorophenyl)diazenyl]-4,5,6,7-tetrahydro-8H-cyclopenta[d]pyrazolo[1,5-a]pyrimidin-8-one
AE-406/41056206	3,3-dichloro-6,8-bisnitro-2,4(1H,3H)-quinolinedione
AE-406/41056481	6-hydroxy-2-methyl-5-(1H-tetraazol-5-ylcarbonyl)-1,2-dihydro-4H-pyrrolo[3,2,1-ij]quinolin-4-one
AE-562/12222164	N-{4,5-bisnitro-1-naphthyl}acetamide

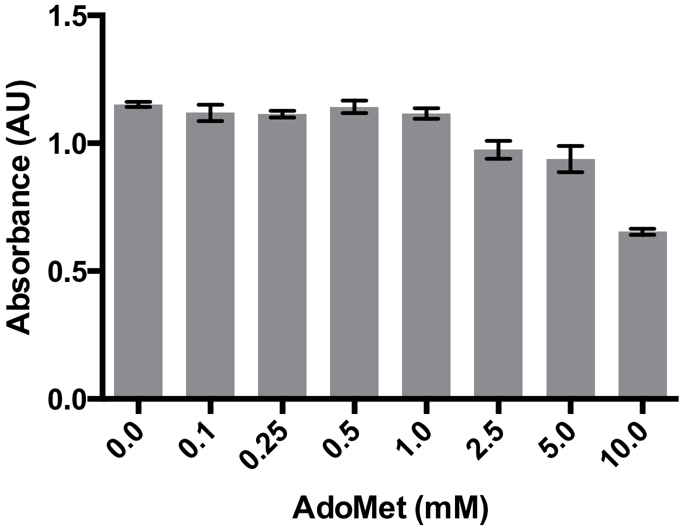

Supplementary Figure S1. The background effect of AdoMet was checked by adding different concentrations of AdoMet in the assay mixture without hAdoMetDC. Concentrations of AdoMet below 1.0 mM had no obvious interference on the assay. The data are shown in means with standard deviations (3 replications).

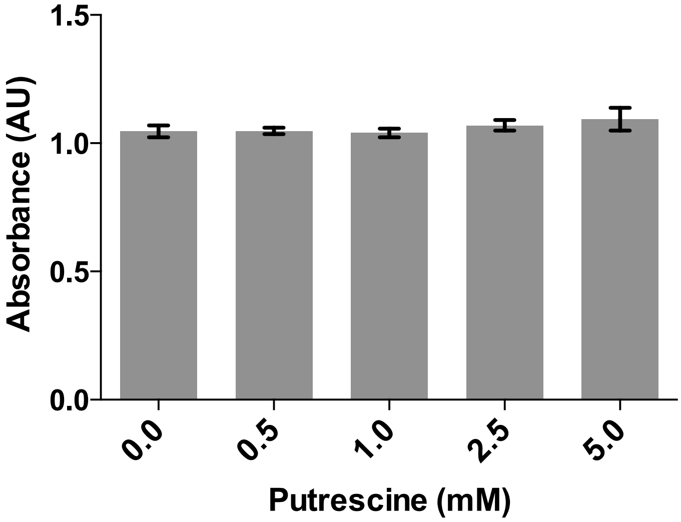

Supplementary Figure S2. The background effect of putrescine was checked by adding different concentrations of putrescine in the assay mixture without hAdoMetDC. At the tested concentrations (0.0-5.0 mM), putrescine had no obvious interference on the assay.

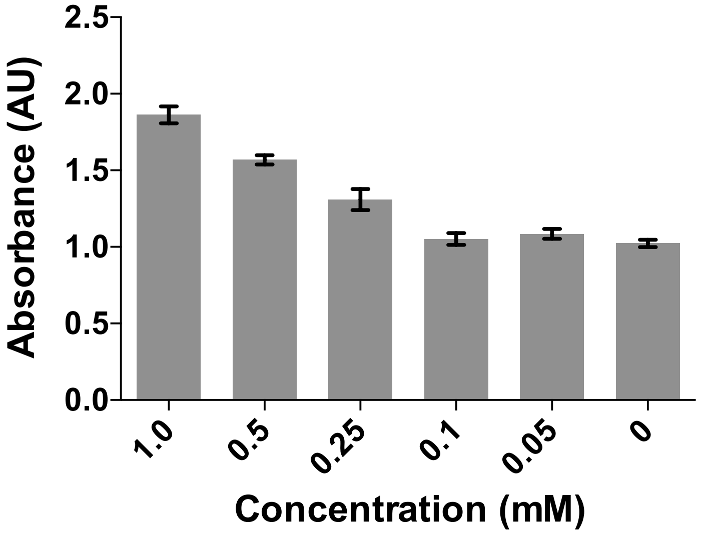

(a)

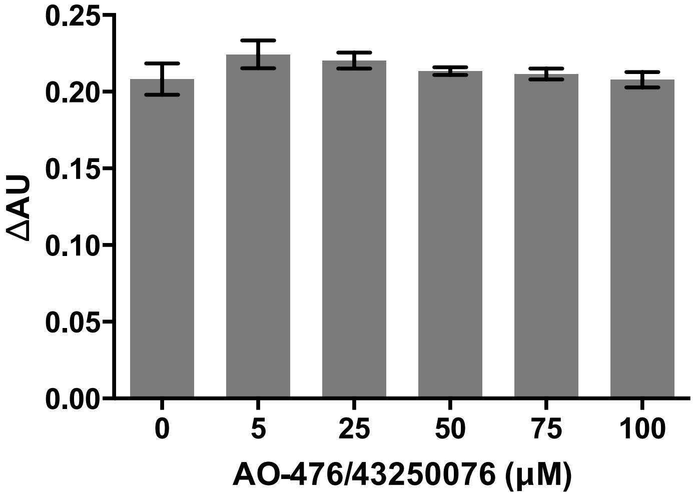

(b)

Supplementary Figure S3. The background effect of MGBG (a) and AO-476/43250076 (b) were checked by adding different concentrations in the assay mixture with the calibration standard (100 μM $NaHCO_3$) instead of hAdoMetDC. Concentrations at 100 μM or lower had no obvious interference on the assay for both. The data are shown in means with standard deviations (3 replications).

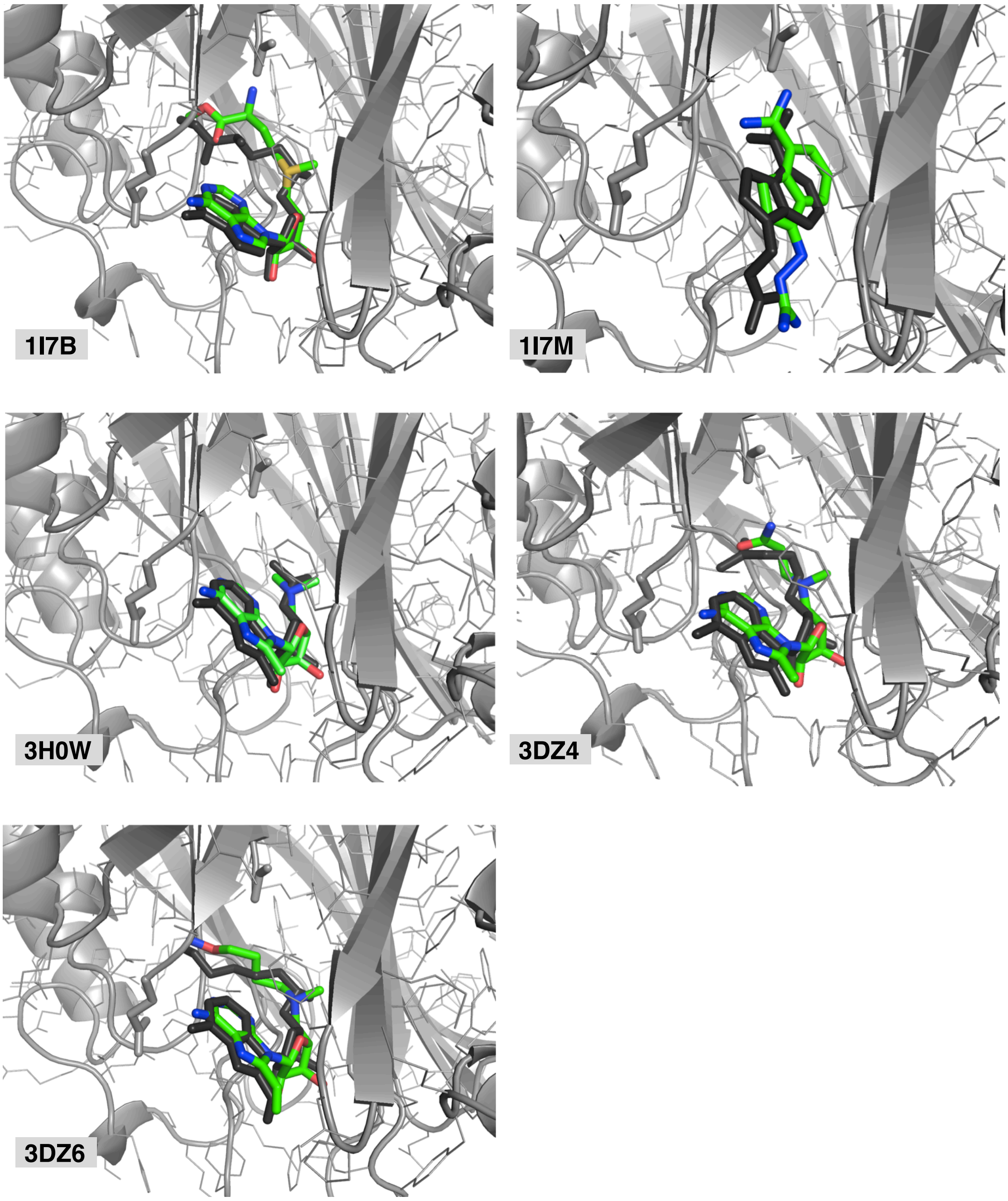

Supplementary Figure S4. The comparison of the docked conformations of known inhibitors and the X-ray conformations. The un-modified active form of hAdoMetDC (PDB ID: 3DZ5) is shown in grey cartoon and used for structure alignment, and the residues 67 and 68 are shown in sticks. The computationally docked models of the known inhibitors are shown in black, and the X-ray conformations are colored by atom. The PDB IDs are marked on the figures for corresponding inhibitors.

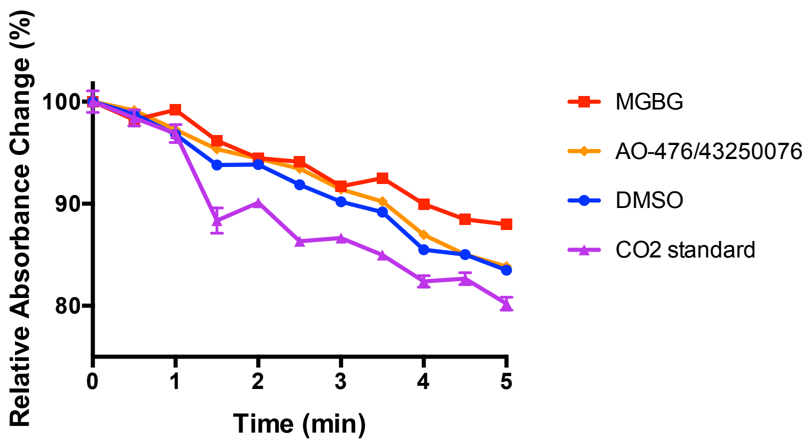

Supplementary Figure S5. Test of a complementary method of AdoMetDC-PEPC-MDH by measuring the fluorescence of NADH. NADH has emission fluorescence at 415 nm when excited by 340-nm light. DMSO is the positive control without inhibitors.
